# Exercise Right Heart Catheterization: The Key to Unraveling the Mystery of Recurrent Syncope

**DOI:** 10.1016/j.jaccas.2025.104890

**Published:** 2025-09-17

**Authors:** Mhd Baraa Habib, Anas Ashour, Mohammed Awad Ashour, Hiba Habib, Waleed K. Abdullatef, Abdulrahman Arabi

**Affiliations:** a Cardiology Department, Heart Hospital, Hamad Medical Corporation, Doha, Qatar; b Internal Medicine Department, Damascus University Hospital, Damascus, Syria

**Keywords:** exercise-induced pulmonary arterial hypertension, exercise right heart catheterization, pulmonary hypertension, systemic lupus erythematosus, vasoreactivity testing

## Abstract

**Background:**

Exercise right heart catheterization (RHC) is a valuable diagnostic tool for identifying hemodynamic abnormalities that may not be evident at rest.

**Case Summary:**

A 36-year-old woman presented with recurrent exertional syncope and dyspnea. Initial resting studies were inconclusive. Resting RHC revealed mild precapillary pulmonary hypertension (PH). However, exercise RHC unmasked a disproportionate increase in mean pulmonary artery pressure during exertion, diagnostic of exercise-induced pulmonary arterial hypertension (EIPAH). Vasoreactivity testing with inhaled nitric oxide demonstrated favorable pulmonary vascular responsiveness. Autoimmune workup confirmed systemic lupus erythematosus. The patient was started on medical therapy and referred to rheumatology and the PH clinic.

**Discussion:**

This case highlights the role of exercise RHC in detecting early pulmonary vascular disease and supports vasoreactivity testing to guide therapy. EIPAH may be the initial manifestation of autoimmune disease and should be considered in young patients with exertional symptoms and nondiagnostic resting evaluations.

**Take-Home Messages:**

Exercise RHC can reveal latent PH when resting measurements are nondiagnostic. In patients with unexplained exertional symptoms, EIPAH should be considered. EIPAH may be the initial manifestation of an underlying autoimmune condition such as systemic lupus erythematosus.

## Graphical Abstract

**Figure:**
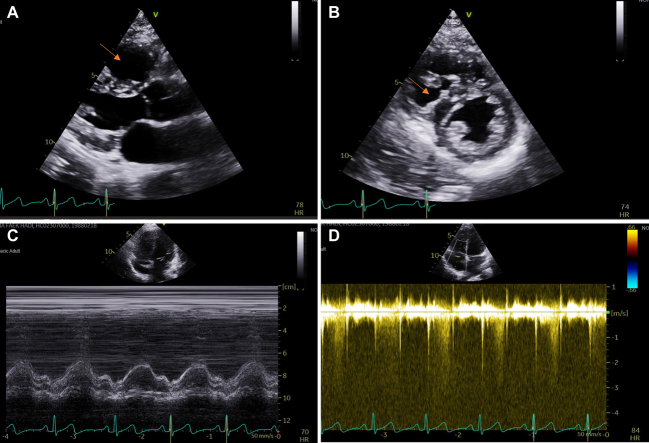

Pulmonary hypertension (PH) is a hemodynamic condition characterized by elevated pulmonary artery pressure (PAP), with significant clinical implications ranging from exertional dyspnea to right heart failure.^1^^,^^2^ In many cases, symptoms precede definitive diagnostic findings on resting studies. Standard right heart catheterization (RHC) at rest may miss early pulmonary vascular disease, particularly in symptomatic patients with borderline or nondiagnostic resting pressures.^3^^,^^4^

Exercise RHC is increasingly recognized as a critical adjunctive tool in evaluating unexplained exertional symptoms. By assessing pulmonary hemodynamics during stress, it can unmask latent pulmonary vascular dysfunction and clarify the pathophysiological basis of exertional symptoms.^3^^,^^4^

We present a case where exertional syncope in a young woman were ultimately explained by exercise-induced pulmonary arterial hypertension (EIPAH), which was not remarkably correlating with resting measurements.

## History of Presentation

A 36-year-old woman presented with several months of exertional dyspnea and recurrent syncope during moderate physical activity. She denied chest pain or orthopnea.

## Past Medical History

She had no prior medical history and was not on any medications.

## Investigations and Management

**Figure 1:**
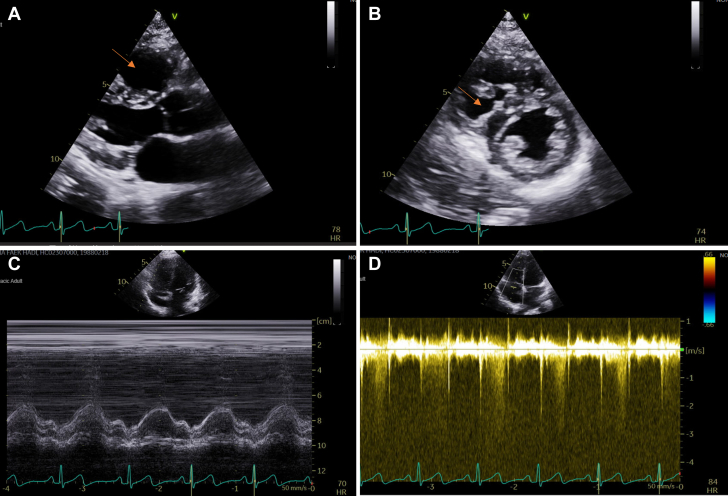
Transthoracic Echocardiographic Images (A, B) A mildly dilated right ventricle (RV) (orange arrow) with mild systolic septal flattening, suggestive of pressure overload. (C) Continuous-wave Doppler signal across the tricuspid valve demonstrating an estimated right ventricular systolic pressure of 53 mm Hg. (D) Assessment of RV function showing a borderline normal tricuspid annular plane systolic excursion of 17 mm. Regional wall motion analysis indicates borderline reduced systolic function at the apex and midsegment of the RV free wall.

**Figure 2:**
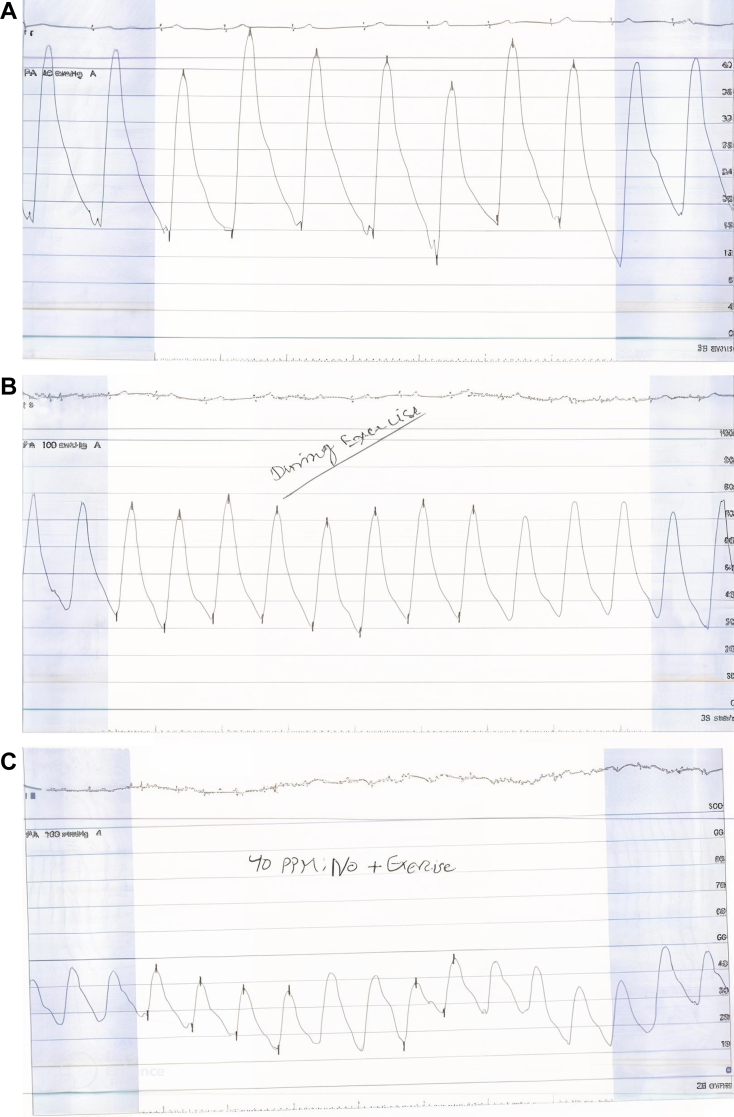
RHC Tracing (A) Right heart catheterization (RHC) tracing at rest. Pulmonary artery pressure (PAP) at rest is 41/16 mm Hg with a mean PAP (mPAP) of 26 mm Hg and heart rate of 61 beats/min. This tracing demonstrates mild elevation in mPAP. (B) RHC tracing during isometric handgrip exercise. PAP increases significantly during exercise to 74/33 mm Hg with an mPAP of 49 mm Hg and heart rate of 82 beats/min. This exaggerated pulmonary pressure response to low-level exertion is diagnostic of exercise-induced pulmonary arterial hypertension. (C) RHC tracing during exercise after administration of 40 parts per million inhaled nitric oxide (iNO). With iNO and continued exertion, PAP markedly improves to 38/20 mm Hg with an mPAP of 29 mm Hg, and the heart rate is 94 beats/min. This blunted pressure response indicates pulmonary vasoreactivity, supporting the use of pulmonary vasodilator therapy.

Initial evaluation revealed normal electrocardiography and chest radiography findings. Transthoracic echocardiography showed a mildly dilated right ventricle (RV) with mildly impaired systolic function (tricuspid annular plane systolic excursion 16 mm), estimated systolic PAP of 52 mm Hg, and preserved left ventricular function (Figure 1). The abnormal echocardiography findings raised immediate concern for PH; therefore, RHC was considered. Resting RHC revealed mild precapillary PH with mean PAP (mPAP) of 27 mm Hg, pulmonary capillary wedge pressure of 10 mm Hg, pulmonary vascular resistance (PVR) of 3.5 Wood units, and a cardiac index of 2.1 L/min/m^2^ (Figure 2A, Table 1).

**Table 1:** Hemodynamic Measurements at Rest, During Exercise, and With Inhaled Nitric Oxide

Measurement	Baseline/Rest	Exercise	Nitric Oxide 20	Nitric Oxide 40 With Exercise
Heart rate (beats/min)	61	69	–	–
PAP (mm Hg): systolic/diastolic/mean	41/16/26	74/33/49	27/17/19	35/17/23
Cardiac output (L/min) (thermodilution)	3.45	4.80	3.62	4.85
Cardiac index (L/min/m^2^)	2.15	2.99	2.25	3.02
PVR (Wood units)	5.5	–	2.7	–

Cardiac index = cardiac output/body surface area; cardiac output measured by thermodilution; nitric oxide administered at 20 parts per million (rest) and 40 parts per million (with exercise); exercise performed via isometric handgrip; PAP = pulmonary artery pressure; PVR = pulmonary vascular resistance.

Given the discordance between symptoms and resting hemodynamics, exercise RHC was performed using handgrip maneuvers. This revealed a pathologic increase in mPAP relative to cardiac output (CO), with an elevated mPAP/CO slope diagnostic of EIPAH (Figure 2B, Table 1).

## Procedural Details

During exercise RHC, inhaled nitric oxide (iNO) was administered. At rest, iNO reduced mPAP and PVR. Repeat handgrip under iNO did not reproduce the hypertensive response seen prior, indicating vasoreactivity (Figure 2C, Table 1).

## Outcome and Follow-Up

Autoimmune workup revealed photosensitivity, positive antinuclear antibody, and anti-Smith antibodies, consistent with systemic lupus erythematosus (SLE). The patient was referred to rheumatology and the PH clinic. Sildenafil 25 mg trice per day and macitentan 10 mg daily were initiated with plans for close follow-up and monitoring of her pulmonary and rheumatologic status. However, the patient went back to her home country and lost her follow-up with us.

## Discussion

Syncope in the context of PH is a red flag symptom. In patients with PAH, syncope—particularly exertional syncope—typically indicates that the CO is not adequately meeting the demands, often due to the severe limitations imposed by the high pulmonary pressures. Mechanistically, syncope in PH is thought to be predominantly hemodynamic in origin rather than due to primary arrhythmias.^5^

This case illustrates how dynamic pulmonary hemodynamic assessment via exercise RHC can bridge the diagnostic gap in patients with unexplained exertional symptoms and inconclusive resting studies. It is not uncommon for the echocardiogram's estimated pulmonary pressures to differ from the values obtained by RHC, and this case appears to illustrate that typical discrepancy. In practice, Doppler echocardiography often provides a reasonable correlation with invasive pressures, but individual readings can deviate substantially.^6^ Echocardiography-derived right ventricular systolic pressure is calculated from the tricuspid regurgitation jet velocity plus an assumed right atrial pressure, and multiple factors can lead to inaccuracies.^7^ If the Doppler signal quality is suboptimal or the tricuspid regurgitation jet is not well aligned, the pressure gradient may be misestimated. The assumption of right atrial pressure (often a fixed value or based on inferior vena cava size) can also introduce error.^7^ Furthermore, hemodynamic conditions can change between the echocardiography and RHC—for instance, volume status, blood pressure, or medications might differ, affecting the pulmonary pressures at the 2 time points.^7^

Standard RHC is performed at rest and may not reflect the full spectrum of pulmonary vascular function. Particularly in young or early-stage patients, symptoms may occur during exertion when pulmonary vascular reserve is overwhelmed. Exercise RHC is designed to assess the behavior of pulmonary pressures and resistance under physiologic stress. It is especially useful when resting mPAP values are borderline (21-24 mm Hg) or symptoms appear disproportionate to resting findings.^1^, ^2^, ^3^, ^4^

Historically, EIPAH was defined as mPAP >30 mm Hg during exercise, but this was abandoned because of poor specificity, especially in older adults. Contemporary criteria focus on the mPAP/CO slope, with values >3 mm Hg/L/min considered pathologic.^8^ This metric reflects an impaired pulmonary vascular response to increased flow, often indicating early pulmonary vascular remodeling. In our case, the steep mPAP/CO slope with handgrip unmasked EIPAH in a patient with borderline resting hemodynamics.

During exercise, PH may develop when the pulmonary vasculature fails to accommodate increased blood flow due to either functional or structural abnormalities. One mechanism involves inappropriate pulmonary vasoconstriction, where the vessels are unable to dilate—or actively constrict—leading to elevated PVR and a subsequent rise in PAP.^9^ In addition, structural remodeling of the pulmonary arteries, such as medial hypertrophy and intimal thickening, reduces vascular compliance and limits the vasodilatory reserve, further contributing to exercise-induced PH.^9^

Although upright cycle ergometry is the standard for exercise RHC, isometric handgrip was used as an alternative due to limited resources. Handgrip increases systemic and pulmonary vascular tone, provoking abnormalities in pulmonary vascular reserve or RV-PA coupling that may go undetected at rest.^10^ When a patient performs a sustained handgrip (clenching a dynamometer or fist for 1-3 minutes), it triggers a surge in sympathetic nervous system activity. This leads to peripheral vasoconstriction and a sharp increase in systemic vascular resistance, which elevates systemic blood pressure significantly. Unlike aerobic exercise, isometric effort raises blood pressure with only a modest increase in heart rate and CO.^11^ In our patient, the handgrip maneuver provided sufficient physiologic stress to reveal a pathologic response. It caused a notable jump in PA pressure, which is probably illustrative of her limited hemodynamic reserve.

PAH is a recognized but uncommon complication of SLE, with prevalence estimates ranging from 0.5% to 14% depending on the population studied.^12^^,^^13^ In many cases, PH arises insidiously due to immune-mediated vascular remodeling, in situ thrombosis, or chronic inflammation affecting the pulmonary arterioles.^14^ Early diagnosis is vital, as delayed treatment leads to RV failure and increased mortality.

In patients with SLE with symptoms suggestive of PH, exercise RHC may provide the first objective evidence of vascular dysfunction. A study by Saggar et al^15^ showed that abnormal hemodynamic responses to exercise in connective tissue diseases may predict progression to resting PH.

Vasoreactivity testing using agents such as iNO helps identify patients who may benefit from pulmonary vasodilator therapy. iNO is a standard agent for acute vasoreactivity testing in PAH. Physiologically, nitric oxide is an endothelium-derived relaxing factor produced in the body to cause vasodilation.^16^ When administered by inhalation at low concentrations (typically 10-40 parts per million), NO selectively diffuses into the pulmonary vasculature and induces potent relaxation of pulmonary arterial smooth muscle by stimulating cyclic guanosine monophosphate production.^16^

Although full vasoreactivity (ie, a >10 mm Hg drop in mPAP to <40 mm Hg with unchanged CO) is rare in connective tissue disease-associated PAH, even partial responsiveness suggests therapeutic benefit from phosphodiesterase-5 inhibitors, endothelin receptor antagonists, or prostacyclins.^17^^,^^18^ Our patient's hemodynamic improvement with iNO supported initiation of sildenafil and macitentan.

The treatment of PH secondary to SLE is typically guided by disease severity and functional class. In early stages (NYHA functional class I or II), immunosuppressive therapy with cyclophosphamide and glucocorticoids may be sufficient. In more advanced cases (NYHA functional class III or IV), a combination of immunosuppression and targeted PH therapy is recommended, with reassessment after 6 months.^13^

Detecting PH early—particularly EIPAH—offers an opportunity for early therapeutic intervention before overt RV dysfunction occurs. SLE-PAH responds variably to immunosuppressive therapy, but outcomes improve when vasodilator therapy is initiated early.^19^^,^^20^ Our patient's case reflects this window for early diagnosis and treatment. Collaboration between rheumatology, cardiology, and PH expert is essential in such cases to ensure comprehensive and coordinated patient care.

## Conclusions

Exercise RHC offers crucial diagnostic insight in patients with unexplained exertional symptoms. Using dynamic assessment, including handgrip as a stress modality and vasoreactivity testing, can uncover early pulmonary vascular disease and enable timely, targeted treatment to improve outcomes.

## Funding Support and Author Disclosures

The authors have reported that they have no relationships relevant to the contents of this paper to disclose.

**Take-Home Messages**

• Exercise right heart catheterization can reveal latent pulmonary hypertension when resting measurements are nondiagnostic. In patients with unexplained exertional symptoms, exercise-induced pulmonary arterial hypertension should be considered.
• Exercise-induced pulmonary arterial hypertension may be the initial manifestation of an underlying autoimmune condition such as systemic lupus erythematosus.
